# Stressful Life Events and Reward Processing in Adults: Moderation by Depression and Anhedonia

**DOI:** 10.1155/2024/8853631

**Published:** 2024-02-03

**Authors:** Sung Min, Raegan Mazurka, Diego A. Pizzagalli, Alexis E. Whitton, Roumen V. Milev, R. Michael Bagby, Sidney H. Kennedy, Kate L. Harkness

**Affiliations:** ^1^Department of Psychology, Queen's University, Kingston, ON, Canada; ^2^Department of Psychiatry, Harvard Medical School & McLean Hospital, Belmont, MA, USA; ^3^Department of Psychiatry, Queen's University, Kingston, ON, Canada; ^4^Providence Care Hospital, Kingston, ON, Canada; ^5^Department of Psychology, University of Toronto, Toronto, ON, Canada; ^6^Department of Psychiatry, University of Toronto, Toronto, ON, Canada; ^7^Centre for Depression and Suicide Studies, St. Michael's Hospital, Toronto, ON, Canada

## Abstract

**Background:**

Exposure to acute stress is associated with reduced reward processing in laboratory studies in animals and humans. However, less clear is the association between reward processing and exposure to naturalistic stressful life events. The goal of the current study was to provide a novel investigation of the relation between past 6-month stressful life events and reward processing, and the extent to which this relation was moderated by depression diagnostic status and state symptoms of anhedonia.

**Methods:**

The current study included a secondary analysis of data from 107 adults (37 current-depressed, 25 past-depressed, 45 never-depressed; 75% women) drawn from two previous community studies. Past 6-month stressful life events were assessed with a rigorous contextual interview with independent ratings. Response to monetary reward was assessed with a probabilistic reward task.

**Results:**

Among current-depressed participants, and among both current- and past-depressed participants with high levels of anhedonia, greater exposure to independent life events outside of individuals' control was significantly associated with poorer reward learning. In direct contrast, among those with *low* levels of anhedonia, greater exposure to independent life events was significantly associated with a greater overall bias toward the more frequently rewarded stimulus.

**Conclusions:**

Results suggest that depression and anhedonia are uniquely associated with vulnerability to blunted reward learning in the face of uncontrollable stressors. In contrast, in the absence of anhedonia symptoms, heightened reward processing during or following independent stressful life event exposure may represent an adaptive response.

## 1. Introduction

A robust preclinical literature has documented that exposure to multiple types of stress in adult animals, including chronic mild stress, chronic social defeat, and repeated, uncontrollable acute stress (e.g., tailshock), is associated with blunted behavioral and neural reward responsivity and motivation [1]. Similarly, in adult humans, exposure to acute laboratory stress challenges (e.g., threat of shock) has been associated with lower striatal and neurophysiological activation in response to, and in anticipation of, reward cues, as well as lower reward learning on behavioral tasks (e.g., [2–4]). Further, recent longitudinal evidence in human adolescent samples suggests that acute stressful life events are associated with blunted reward positivity (RewP)—an event-related potential component indexing reduced reward processing [5, 6].

However, to date, there has been almost no research examining the relations between reward processing and life events in individuals' natural, day-to-day environments—fights and arguments with family members and coworkers, rejections from friends and romantic partners, job disappointments, health scares, etc. Are these sorts of common, proximal, and idiographic stressors associated with the same alterations in reward processing as seen in the context of contrived stressors in highly controlled laboratory environments, or following major developmental traumas (e.g., childhood sexual abuse; [7])? Further, are particular stressful life events more strongly associated with alterations in reward processing than others?

The current study presents a novel examination of the relation of acute life events experienced in the past 6 months to reward processing in a community sample of adults. We assessed reward processing with the probabilistic reward task (PRT; [8]), which allowed us to examine both “reward learning”—defined as the ability to integrate information about reinforcement history over time to optimize future responses—and “response bias”—defined as the general tendency to selectively choose a rewarded stimulus. In preclinical studies examining the relation of acute recent stress to reward processing assessed with the animal version of the PRT, similar patterns of association are observed across reward learning and response bias (see [9]). In the current study, we similarly hypothesized that greater exposure to life events in the past 6 months would be significantly associated with lower performance across both reward indices.

We used a rigorous interview method to assess, code, and rate stressful life events that included contextual ratings of life event occurrence and severity by independent judges, as well as the ability to address domains of stress of most theoretical relevance to reward processing. This methodology is particularly important in the current design as it minimizes potential negative biases in event recall that could result in a spurious association between stress and reward processing (see [10]). A key distinction is between life events that are independent of the individual's behavior (i.e., uncontrollable events, such as job layoff due to factory closure or child's cancer diagnosis) versus events that are at least in part dependent on the individual's behavior, either in the interpersonal (e.g., romantic relationship breakup) or noninterpersonal (e.g., fired from a job due to negligence) domains.

Both independent and dependent life events are significantly causally associated with the onset of depression [11, 12]. However, in terms of associations with reward processing, the strongest conclusion is that exposure to recent stressors that are uncontrollable and, thus, *independent* of behavioral agency is broadly associated with *lower* reward sensitivity (i.e., consummatory anhedonia), reward learning, and reward-related decision-making (i.e., motivational anhedonia). In animal models, uncontrollable stress (e.g., tailshock) is associated with passive coping strategies aimed at conserving energy and minimizing risk, which are related to inhibited release of mesolimbic dopamine (see reviews by [13, 14]). In human studies, exposure to uncontrollable and unpredictable stressors (e.g., childhood sexual abuse; threat of shock in laboratory paradigms) is associated with a lower motivation to pursue rewards (see [9]). Thus, we hypothesize here that greater exposure to independent life events in the past 6 months would be significantly associated with lower reward processing.

At the same time, *social* stress (e.g., chronic social defeat) in animal models is also significantly associated with lower reward sensitivity, reward learning, and motivation to work for reward (e.g., [15]). Further, in human studies of adolescents, *interpersonal* stress (e.g., relational bullying) is more strongly associated with blunted reward sensitivity than other nonrelational stressors (e.g., [16]). Indeed, reduced motivation to pursue and learn from rewards may be an adaptive strategy in the face of interpersonal stress as a means of defusing interpersonal conflict and preventing rejection, which would have been catastrophic in humans' early evolutionary environment [17]. Therefore, we also hypothesized here that greater exposure to dependent-interpersonal life events in the past 6 months would be significantly associated with reduced response bias and reward learning.

Our sample comprised three groups: individuals in a current episode of a unipolar depressive disorder, individuals with a past history of depression, and individuals with no history of depression. There is meta-analytic evidence that the state of depression and high-state symptoms of anhedonia, in particular, are associated with reduced behavioral, neurophysiological, and neurofunctional responsivity to rewarding stimuli, as well as reduced motivation to work for, and learn from, reward [18]. Moreover, low reward processing has been proposed as a trait marker of depression that distinguishes even those in remission from never-depressed individuals (e.g., [19–21]), particularly in the context of remission from melancholic depression, which is characterized by high levels of anhedonia [22]. However, the evidence bearing on this is mixed, with a further study showing no significant difference between those with depression in remission and never-depressed groups [23].

To our knowledge, the current study is unique in examining whether depression status and anhedonia symptoms differentially *tune* the relation between stress exposure and reward processing. That is, the current design afforded us with the novel opportunity to examine diagnostic status and state anhedonia as *moderators* of the relation between recent, acute stressful life events and reward processing. We hypothesized that the relations between stressful life event exposure and reward processing would be significantly stronger in those with a current or past diagnosis of depression versus in never-depressed individuals. In secondary analyses, we also tested the hypothesis that the relation between stressful life events and reward processing among those with a current or past history of depression would be significantly stronger among those with more severe state symptoms of anhedonia.

## 2. Method

### 2.1. Participants

The current study involved secondary analysis of data from 107 adults drawn from two previous studies of stress and depression in adults aged 18-65 (Study 1 *n* = 64; 66% women; 75% White; *M* age = 33.84, SD = 15.15; [24]) and young adults aged 18-29 (Study 2 *n* = 43; 65% women; 79% White; *M* age = 19.88, SD = 2.29; [25]). All participants in the current report were recruited from community advertisements. Separate advertisements targeted adults with versus without current symptoms of depression. All procedures were approved by the Health Sciences Research Ethics Board at Queen's University, and participants provided written, informed consent. Inclusion criteria for the depression groups in both studies were meeting the Diagnostic and Statistical Manual for Mental Disorders (DSM-IV; [26]) criteria for either a current (*n* = 37) or a past (*n* = 25) unipolar depressive disorder. Exclusion criteria for both studies were lifetime diagnosis of bipolar disorder, psychotic disorder, alcohol or substance dependence, or medical disorder that could cause depression. Participants in the never-depressed group (*n* = 45) could not meet current or lifetime criteria for any psychiatric diagnosis. Further, participants were included in the current report if they completed the life event interview and the reward task (described below). Additional clinical characteristics are listed in Table 1, stratified by depression status.

Given that this was a secondary analysis of data collected as part of two previous studies, an *a priori* power analysis was not conducted for the current research question. However, post hoc sensitivity analysis indicated that the current sample size of 107 had 80% power to detect a small effect (0.10) and 95% power to detect a medium effect (0.20) in a multiple regression with five predictors.

Participants in Study 1 were significantly younger, on average, than those in Study 2 (*t*(67.24) = 7.25, *p* < .001). Participants in Study 1 and Study 2, however, did not differ significantly on any other demographic characteristic or on any of the life event or reward task variables (all *p*s > .07). However, there were some differences for participants across studies on some clinical-based variables; compared to Study 2, Study 1 included more participants with current depression (44% vs. 21%, *χ*(2) = 7.37, *p* = .03) and correspondingly higher scores in Hamilton Rating Scale for Depression (HRSD; [27]) (*M*s = 9.64, 4.42; SDs = 9.41, 5.85, *t*(105) = 3.54, *p* < .001) and in Anhedonic Depression subscale (AD) (*M*s = 69.29, 58.86; SDs = 19.48, 16.87, *t*(105) = 2.94, *p* = .002) of the Mood and Anxiety Symptom Questionnaire (MASQ; [28]). In preliminary model building, we determined that results were sufficiently robust to including age and study (1 vs. 2) as covariates and data were collapsed across studies and are presented without these covariates for ease of interpretation. Models controlling for the study are presented in Supplemental Tables 1 and 2.

### 2.2. Measures

#### 2.2.1. Depression Diagnoses and Symptoms

Current and past history of Axis I disorders was determined with the Structured Clinical Interview for DSM-IV Axis I Disorders (SCID-I/P; [29]) in both Study 1 and 2. The DSM-IV criteria were used instead of DSM-5 because data collection started before the publication of the SCID-5 [30]. In Study 1, current diagnostic status was also determined with the SCID-I/P, whereas, in Study 2, the Longitudinal Interval Follow-up Evaluation (LIFE; [31]) was used to confirm the diagnostic status at the time of completion of the PRT. In both studies, the level of depression symptoms at the time of completion of the PRT was assessed with the 17-item clinician-rated HRSD. All interviews were administered by senior graduate students in clinical psychology or licensed clinical psychologists who were trained to reliability by the senior author (see [32]).

Of the 37 participants in the current depression group, depressive disorder diagnoses included major depressive disorder (*n* = 34) and depressive disorder not otherwise specified (*n* = 3). The 25 participants in the past-depressed group did not meet the current criteria for even a subthreshold depression diagnosis but had met the criteria for a depressive disorder at some time in the past (major depressive disorder (*n* = 17); dysthymic disorder (*n* = 3); adjustment disorder with depressed mood (*n* = 3); or depressive disorder not otherwise specified (*n* = 2)). Comorbid diagnoses and other clinical characteristics are presented in Table 1.

Symptoms of anhedonia were assessed at the time of completion of the PRT with the AD subscale of the MASQ. The 22-item AD subscale is a measure of low positive emotionality and includes items consistent with anticipatory anhedonia (e.g., “looked forward to things with enjoyment” reverse-coded), consummatory anhedonia (e.g., “felt like there wasn't anything interesting or fun to do”), and motivational anhedonia (e.g., “felt like it took extra effort to get started”). Participants were asked to indicate how much they had experienced each symptom in the past seven days on a Likert scale ranging from “1” (not at all) to “5” (extremely). Factor analysis of the MASQ AD subscale suggests the existence of a strong general factor that is stable over time [33].

#### 2.2.2. Stressful Life Events

The Life Events and Difficulties Schedule (LEDS-II; [34]) is a semistructured, contextual interview that queries about multiple different life events in several domains (i.e., health, housing, finances, education/occupation, marital/partner relationships, other relationships (e.g., confidants and other family members), crime/legal, and crises). Senior clinical psychology graduate student interviewers were trained specifically not to query about participants' subjective perceptions of events. Interviews were recorded and subsequently written up into vignettes by research assistants who were trained to delete any mention of subjective responses. The vignettes were then presented to, and rated by, a team of 2-4 research assistants who were unaware of participants' diagnoses or responses on the PRT. Ratings were based on the LEDS-II manual, which includes detailed rules and criteria for rating as well as over 5000 exemplars to anchor the ratings. The severity of each event was rated on a 5-point scale (1 = *marked*, 2 = *high moderate*, 3 = *low moderate*, 4 = *some*, and 5 = *little/none*). Raters also determined whether each event was independent or at least in part dependent on the participants' agency. Dependent events were further categorized as either interpersonal or noninterpersonal. Pairwise comparisons among raters for threat ranged from *k* = .74 to *k* = .78 and for independence were *k* = 1.00. Discrepancies among raters were resolved through discussion, and the consensus ratings for all scales were used in analyses.

The life events included in the current analyses were those experienced in the 6-month period prior to the completion of the reward task (described below). We chose 6 months because we were specifically interested in acute events experienced proximal to the assessment of reward (see [35]). For the purposes of analysis, severity scores of each event experienced during this 6-month period were reverse-coded and summed over the time period. Separate sums were calculated for independent, dependent-interpersonal, and dependent-non-interpersonal events. Means and standard deviations for the life event variables stratified by depression group are presented in Table 2. Frequencies and percentages of participants reporting at least one event of each type are presented in Supplemental Table 3.

#### 2.2.3. Reward Processing

Reward processing was assessed with the probabilistic reward task (PRT; [8]). The PRT measures participants' ability to modulate their behavior toward monetary rewards based on reward history. In this computerized task, participants were presented with a schematic face with two dot eyes and a straight-line mouth and were asked to identify, by key press, whether the mouth was short (11.5 mm) or long (13 mm). Stimuli were presented over three blocks of 100 trials each. A response bias was elicited by implementing an asymmetrical reinforcement schedule, where correct identification of the long mouth (“rich stimulus”) was rewarded three times more frequently than correct identification of the short mouth (“lean stimulus”). In each block, only 40 correct trials (30 rich, 10 lean) were followed by a reward (“Correct!! You won 5 cents”). Participants were told at the outset that not all correct responses would be rewarded, but they were unaware of the asymmetrical reinforcement schedule. The tendency for participants to develop a bias toward selecting the rich stimulus was taken as evidence of the ability to use past reward experience to motivate future behavior.

Trials with reaction times less than 150 ms or longer than 2500 ms were excluded. After this first exclusion, trials with reaction times (following natural log transformation) falling +/– 3 SD away from the mean were excluded. Following prior work [36], response bias was calculated as Log *b* = 1/2 log (((Rich_correct_ + 0.5)^∗^ (Lean_incorrect_ + 0.5))/((Rich_incorrect_ + 0.5)^∗^ (Lean_correct_ + 0.5))). High response bias indicates high rates of correct identification (hits) for the rich stimulus, and high miss rates for the lean stimulus. Our two primary metrics of interest were (a) response bias—average response bias across the three blocks of trials—and (b) reward learning—participants' response bias score in block 3 minus their response bias score in block 1 [36]. The correlation between response bias and reward learning was *r* = .60.

### 2.3. Procedure

Procedures for the original studies from which participants were drawn differed slightly [24, 25]. In Study 1, participants came into the lab to complete the diagnostic and symptom measures. To relieve participant burden, the PRT and LEDS were completed at a second session approximately two weeks later. In Study 2, participants also completed the diagnostic and symptom measures at a first session, and then the PRT at a second session approximately two weeks later. However, in Study 2, the LEDS was completed at a follow-up session approximately six months later. The *total* time period covered by the LEDS in both Study 1 and Study 2 was the 12-month period prior to the LEDS interview. However, only life events experienced in the 6-month period prior to the completion of the PRT were included in the current analyses (see above). Despite variations in procedure, we note that recall for life events is consistent across time [37], and the inclusion of study (1 vs. 2) as a covariate did not alter the pattern of results presented below (see Supplemental Tables 1 and 2).

### 2.4. Data Analysis

Our primary research question was assessed through two multiple moderated regression analyses in R [38]. The PRT parameters (reward learning or response bias) served as the dependent variables in separate models. The three stressful life event variables (independent events, dependent-interpersonal events, and dependent-non-interpersonal events) were entered together as the independent variables in all models. The depression group variable (dummy coded: current-depressed group (1, 0), past-depressed group (0, 1), and never-depressed group (0, 0)) was entered in each model as the moderator [39]. Two *a priori* contrasts were specified comparing each of the current-depressed and past-depressed groups to the never-depressed group. Significant life events by group contrast interactions were followed up with simple slopes examining the relation of life events to reward processing stratified by group.

Our secondary research question was assessed in the subsample of participants with a current or lifetime diagnosis of a depressive disorder, again through multiple moderated regression analyses in R [38]. The PRT parameters (reward learning or response bias) served as the dependent variables in separate models. The three stressful life event variables (independent events, dependent-interpersonal events, and dependent-non-interpersonal events) were entered together as the independent variables in all models, and AD scores were entered in each model as the moderator. Significant life events by AD interactions were followed up with the Johnson-Neyman (J-N) technique [40] in PROCESS [39]. This technique identifies “regions of significance” for the moderator (AD scores) at which the relation between the independent variable (life events) and the dependent variable (reward learning or response bias) becomes significant [41].

## 3. Results

### 3.1. Descriptive Characteristics

Descriptive characteristics of the sample are provided in Table 1, stratified by depression group. Groups did not differ significantly in gender, ethnicity, or socioeconomic status (SES; as per the Hollingshead Index of Social Position [42]). However, the current-depressed group was significantly older than the never-depressed group (*t*(63.53) = 2.27, *p* = .03). Further, as expected, the current-depressed group scored significantly higher on the HRSD than the past-depressed group (*t*(58.94) = 9.41, *p* < .001), which scored significantly higher than the never-depressed group (*t*(27.57) = 5.15, *p* < .001). Moreover, the current-depressed group reported significantly higher AD scores than the past-depressed group (*t*(47.36) = 6.82, *p* < .001), which scored higher than the never-depressed group (*t*(38.98) = −5.39, *p* < .001). The current- and past-depressed groups did not differ significantly in age of first depression onset, current antidepressant medication use, or the presence of a current comorbid Axis I disorder (all *p*s > .34). However, the current-depressed group had a significantly higher number of lifetime depressive episodes (see Table 1).

Average scores of reward learning were positive (see Table 2), suggesting that, overall, participants demonstrated learning about reinforcement history over time to optimize future responses. Neither reward learning nor response bias was significantly associated with gender, age, ethnicity, or SES (all *p*s > .36). Further, within those with a current or past diagnosis of depression, neither reward learning nor response bias was significantly associated with antidepressant medication use, the presence of a comorbid disorder, or age of first depression onset (all *p*s > .10). However, a greater number of lifetime depressive episodes was significantly associated with lower reward learning (*r*(59) = −.27, *p* = .03). All of our primary models were robust to the inclusion of gender, age, ethnicity, SES, and, in depression subgroup analyses, number of depressive episodes. Therefore, the models without these covariates are presented below for ease of interpretation.

### 3.2. Relation of Life Events to Reward Learning

Descriptive statistics of the primary study variables by depression group are provided in Table 2. Groups did not differ significantly on any of the life event or reward processing variables.

Regression parameters for the model with reward learning are provided in Table 3. As indicated, the relations of both independent life events and dependent-non-interpersonal life events to reward learning were significantly moderated by depression group. Contrary to hypotheses, the relation of dependent-interpersonal life events to reward learning was not significant, either on its own or in interaction with depression group.

**Figure 1:**
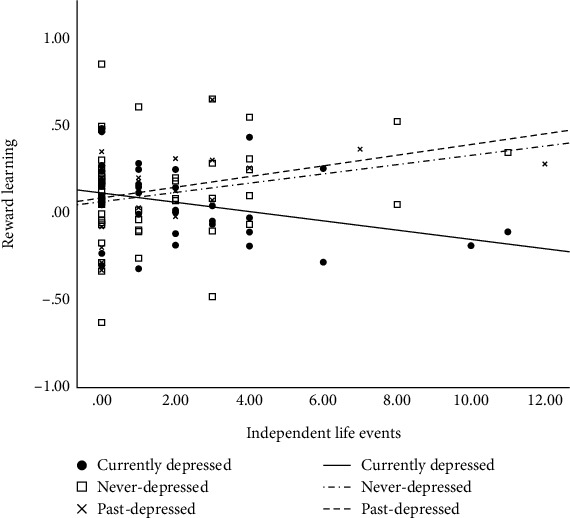
The relation between independent life events and reward learning stratified by depression group.

For independent life events, the *a priori* contrast analysis revealed that the relation of life events to reward learning differed significantly between the current-depressed and never-depressed groups (see Table 3). In contrast, the past-depressed and never-depressed groups did not differ significantly. As hypothesized, in the current-depressed group, greater exposure to independent life events was significantly associated with poorer reward learning (*b* = −.027, *t*(35) = −2.11, *p* = .04) (see Figure 1). In contrast, among the past-depressed and never-depressed individuals, the direction of the associations between independent life events and reward learning was positive, but the associations were not statistically significant (*p*s > .08).

**Figure 2:**
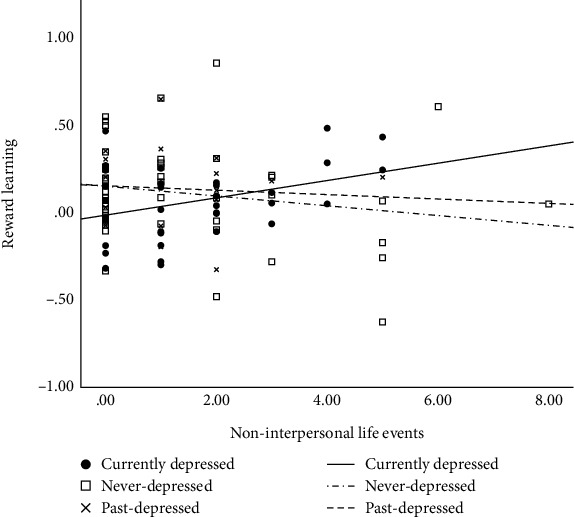
The relation between noninterpersonal life events and reward learning stratified by depression group.

For dependent-non-interpersonal life events, again, the *a priori* contrast analysis revealed that the relation of life events to reward learning differed significantly between the current-depressed and never-depressed groups, whereas the past-depressed and never-depressed groups did not differ significantly (see Table 3). However, in direct contrast to the results reported above, in the current-depressed group, greater exposure to dependent-non-interpersonal life events was significantly associated with *better* reward learning (*b* = .05, *t*(35) = 2.21, *p* = .03). In contrast (see Figure 2), no evidence was found for a significant association between dependent-non-interpersonal life events and reward learning in either the past-depressed or never-depressed groups (*b*s < −.01, *t*s < −0.35, *p*s > .16).

#### 3.2.1. Moderation by Anhedonia

In secondary analyses, we examined whether the relation between independent and noninterpersonal life events to reward learning in the current- and past-depressed groups was significantly moderated by symptoms of anhedonia. In both models we controlled for severity of overall depressive symptoms (HRSD scores), allowing us to investigate the specific impact of anhedonia above and beyond the general distress associated with depression.

**Figure 3:**
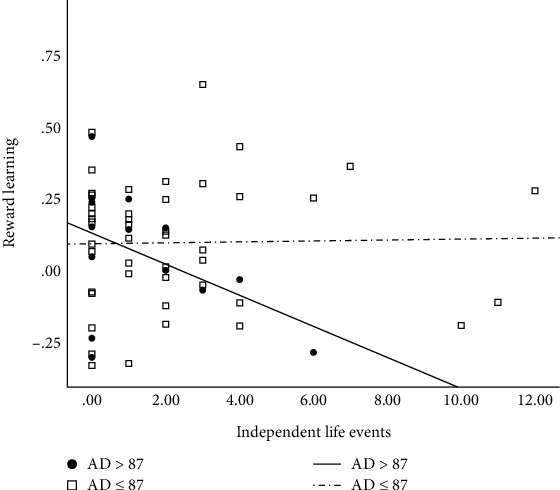
Conditional effects of independent life events on reward learning for Johnson-Neyman-identified regions of significance in anhedonic depression scores in current- and past-depressed group (*n* = 62). Note: AD = Mood and Anxiety Symptom Questionnaire Anhedonic Depression Subscale scores.

The overall model for independent life events approached significance (*R*^2^ = .14, *F* (4, 56) = 2.28, *p* = .07). The interaction between independent life events and AD scores was significant (*b* = −.001, *t*(56) = −2.42, *p* = .019). Follow-up analyses using the J-N method revealed that greater exposure to independent events was significantly associated with *poorer* reward learning, but only among those with higher levels of anhedonia (MASQ AD scores > 87; 25.8% of the sample with current- or past-depression; all *p*s < .05; see Figure 3). In contrast, there was no evidence of a significant association between independent life events and reward learning among those with lower severity of anhedonia (MASQ AD scores ≤ 87; 74.2% of the sample with current- or past-depression; all *p*s > .058).

The overall model for dependent-non-interpersonal life events was not significant (*R*^2^ = .09, *F* (4, 56) = 1.41, *p* = .24). Further, the interaction between noninterpersonal life events and AD scores did not approach significance (*b* = .0006, *t*(56) = 0.398, *p* = .692).

### 3.3. Relation of Life Events to Response Bias

In preliminary model building, we determined that depression group did not significantly moderate the relation of any of the life event variables to response bias (all *ps* for the moderation parameters > .25). Therefore, the parsimonious model excluding the interaction terms is shown in Table 4. The overall model was significant. Contrary to hypotheses, neither dependent-interpersonal nor dependent-non-interpersonal life events were significantly associated with response bias. Further, the relation of independent life events and response bias was *positive*; that is, higher levels of independent life events were significantly associated with a *higher* response bias.

#### 3.3.1. Moderation by Anhedonia

**Figure 4:**
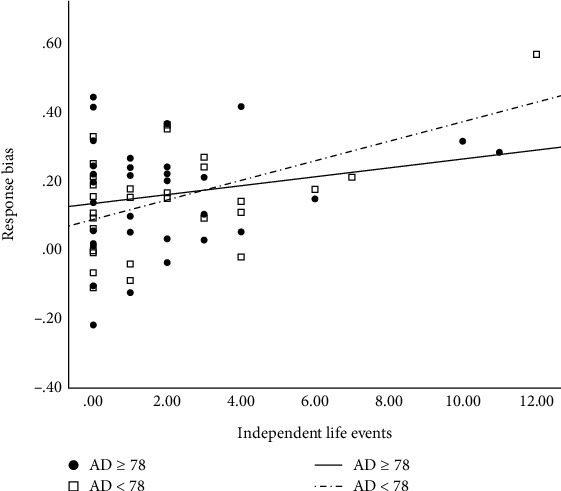
Conditional effects of independent life events on response bias for Johnson-Neyman-identified regions of significance in anhedonic depression scores in current- and past-depressed groups (*n* = 62). Note: AD = Mood and Anxiety Symptom Questionaire Anhedonic Depression Subscale scores.

In secondary analyses, we examined whether the significant positive relation between independent life events and response bias was significantly moderated by symptoms of anhedonia in the current- or past-depressed groups. The overall model was significant, (*R*^2^ = .19, *F*(3, 57) = 4.43, *p* = .01), and the interaction term was also significant (*b* = −.0008, *t*(60) = −2.12, *p* = .04). The J-N follow-up results revealed that, as above, higher levels of independent events were significantly associated with a higher response bias. However, this was only the case among those with *lower* levels of anhedonia (MASQ AD scores < 78.00; 46.77% of the sample with current- or past-depression; all *p*s < .023; see Figure 4). In contrast, there was no evidence for a significant association between independent life events and response bias in those with higher levels of anhedonia (MASQ AD scores ≥ 78.00; 53.23% of the sample with current- or past-depression; all *p*s > .05).

## 4. Discussion

In the current study, we provided novel evidence for an association between exposure to stressful life events and performance on a behavioral measure of reward processing. The exact patterns of association were complex and depended upon the type of reward processing examined (reward learning versus response bias) and the type of life event exposure. A general finding, however, was that *current*-depressed participants stood out as having distinct patterns of association from never-depressed individuals, whereas those with past depression showed patterns that were nearly identical to those of the never-depressed group. These results suggest, therefore, that the relation between stress and reward processing seen in the state of depression may normalize (i.e., approximate that seen in the never-depressed group) upon remission. Within-subject studies that follow individuals from acute episode to remission are required to substantiate this suggestion.

Consistent with hypotheses, among those in the current depression group, greater exposure to life events that were largely outside of individuals' control and *independent* of their actions was significantly associated with poorer reward learning. This finding in our currently depressed group is consistent with preclinical findings showing that uncontrollable stress negatively affects the ability to learn from reward (e.g., [43]). Furthermore, it suggests that alterations in reward processing may not be limited to exposure to childhood trauma or contrived laboratory stressors but also extends to a wide variety of day-to-day proximal life events. The qualitative descriptions of the independent life events experienced by the current-depressed individuals in our sample reveal the unpredictable and uncontrollable nature of these exposures (details have been altered slightly to preserve confidentiality). For example, one respondent was evicted from their apartment unexpectedly because the landlord chose to sell the property, and the respondent could not afford a new apartment. Another individual unexpectedly learned that their grandmother had only six weeks to live. When the grandmother passes, the respondent will have to assume care of their grandfather who has a chronic neurodegenerative disease. A third respondent learned of a best friend's sudden and unexpected death by suicide.

The specific reasons for the dissociation in the directionality of the relation between stress exposure and reward processing in the current-depressed versus the past- and never-depressed participants in the current study are unclear and require further investigation. This dissociation is unlikely to be accounted for by clinical characteristics, such as the presence of comorbid psychopathology or treatment history, because the current- and past-depressed groups did not differ significantly in terms of these characteristics. However, in secondary analyses, we found that the significant negative relation between independent life event exposure and reward learning in both the current- and past-depressed groups was specific to those with high levels of anhedonia, even after controlling for the overall level of depression symptom severity. Therefore, it is possible that the severe symptoms of anhedonia, in particular, serve as an additional stressor that overwhelms individuals' cognitive capacity to learn from reward [44]. Prospective studies that follow individuals from episode to remission are required to determine whether, and what, specific features of depressive psychopathology are responsible for the negative association between acute, uncontrollable life event exposure and reward learning. It is also possible that the current-depressed individuals, and the current- and past-depressed individuals with high anhedonia, differed from those low in anhedonia and the never-depressed individuals on additional factors that could have heightened their stress load and, hence, raised vulnerability to blunted reward learning in the face of independent life events. Although it was beyond our scope to examine these variables in the current study, factors such as a background context of chronic stress and a history of childhood maltreatment have been associated with blunted reward learning in previous studies (see [9, 45]) and could serve as additional moderators of the current associations.

In direct contrast, in the full sample and, in particular, among those with *low* levels of anhedonia, there was a *positive* association between exposure to independent life events and response bias. That is, higher levels of independent life event exposure were significantly associated with a *greater* ability to develop a bias in favor of the more frequently rewarded stimulus. One potential explanation for this positive association may be that in the context of low depressive psychopathology, exposure to acute, uncontrollable life events may signal to the individual the need to be vigilant, thus heightening reward sensitivity (i.e., bias to pursue reward) as an adaptive response. Indeed, an important function of the neurophysiological stress system (i.e., the hypothalamic-pituitary-adrenal (HPA) axis) is to mobilize the psychological and physiological resources that enable active coping [46]. In animal models, exposure to acute stressors is associated with corticotropin releasing factor- (CRF-) mediated *increases* in extracellular dopamine in the nucleus accumbens and medial prefrontal cortex, which promotes reward sensitivity and cognition (e.g., [47]). Further, HPA axis-mediated emotional and physiological arousal because of acute stress in adult animals has also been associated with heightened neurogenesis and dendritic architecture in the hippocampus (e.g., [48, 49]), as well as improvements in cognition and memory [50].

An important strength of the current study was its focus on naturalistic life events in individuals' day-to-day environments, potentially maximizing the external validity and generalizability of the results; however, the lack of control inherent in such design comes at the expense of internal validity, such that the precise direction of the association between stressful life event exposure and reward processing cannot be determined. A complementary (although not mutually exclusive) explanation for the above positive association between stressful life events and reward processing is that individuals with tendencies toward higher reward processing may be more likely to engage with the environment, thus resulting in greater exposure to, and even generation of, life events [51]. We believe that this explanation could help to account for the unexpected positive association between dependent, noninterpersonal life event exposure and reward learning in our current-depressed group. Dependent life events are, by definition, at least in part caused by the actions of the individual, and thus, depressed individuals who are engaging with the environment would be expected to have higher levels of these exposures. For example, several of the individuals in the currently depressed group reported multiple life changes in the 6-month study time period that required initiative and planning, such as starting new jobs or academic programs, moving to a new city, and taking qualifying examinations. Prospective, longitudinal studies with multiple time points are needed to fully clarify the potential bidirectional and transactional nature of the relation between these sorts of dependent-non-interpersonal stressful life events and reward processing, as well as the specific clinical characteristics such as anhedonia that may facilitate or inhibit these associations (e.g., [52]).

Contrary to hypotheses, there was no evidence for an association between dependent-interpersonal events and either reward learning or response bias. This stands in contrast to theoretical work positing a specific association between interpersonal stress and anhedonia [17]. One possible explanation for the lack of a significant association in the current sample is that the frequency and severity of interpersonal life events were low. For example, of the 141 dependent life events reported in the 6-month time frame across all participants, only 41 (29%) were rated as interpersonal, and only seven of these 41 (17%) were rated as “marked” or “high moderate” in severity. Previous research findings and theory have singled out *rejection* (e.g., romantic partner terminates the relationship; sister stops speaking to the respondent) as the specific interpersonal stressor that may be most strongly associated with anhedonia [53]. Future research specifically focusing on samples with high rates of rejection events are required to provide a stronger test of the relation between interpersonal stress and reward processing.

Our findings from this investigation should be interpreted in the context of the certain limitations associated with the study design. First, our sample was small, ethnically homogenous, and consisted of volunteers from the community. Thus, replication in a larger and more diverse sample representative of the population of individuals with unipolar depression is necessary. Specifically, a larger sample would enable us to investigate the association with reward processing of even more specific types of life stress, such as interpersonal loss, which have shown specific relations to depression outcomes (e.g., [54]). Second, the cross-sectional and observational design of our study does not allow us to determine the directionality of the relations among reward processing, life stress, and depression. Third, our conceptualization of reward was necessarily narrow given the context of the study design. Future investigations are needed to generalize the current findings using a broader range of reward constructs assessed at different and preferably multiple levels (e.g., neurofunctional, neurophysiological, and behavioral).

Finally, the LEDS relies on retrospective reporting of life events and thus may be subject to recall bias. For example, it is possible that individuals with blunted reward processing may be more likely to report stressful life events, which, given the cross-sectional design, could provide an alternative explanation for our findings. While we cannot fully rule out recall bias as an alternative explanation for our findings, it is important to note that the LEDS is specifically designed to minimize such biases through the use of a contextual interview with multiple probes that prime autobiographical memory; moreover, independent judges rate the presence, severity, and domain of life events according to anchored, manualized criteria that enhance even further the integrity and quality of life event data, which is by nature subjective. The contextual method of life event assessment has shown superior reliability and predictive validity to traditional life event checklists (see [10]).

In sum, greater exposure specifically to independent (i.e., uncontrollable) life events in the past six months was significantly associated with evidence of poorer learning from reward. However, this relation was specific to those individuals who were either currently in an episode of depression or who had a lifetime history of depression and high-state symptoms of anhedonia. In contrast, among never-depressed individuals, and among those with a lifetime history of depression currently experiencing low levels of anhedonia, greater independent life event exposure was significantly associated with a *stronger* bias to reward. In combination, these results support the conclusion that stress exposure and reward processing are related and that the relation between stress and reward extends to the types of idiographic acute life events that individuals encounter in their naturalistic environment. Our results also suggest that there is something unique about the episode of depression and high-state symptoms of anhedonia that may strengthen the association between uncontrollable stress and blunted reward learning. An important translational direction emerging from the current results is to determine whether treatments that target dopamine-mediated reward learning systems, such as behavioral activation [55] or dopamine agonist augmentation (e.g., aripiprazole; [56]), may show greater efficacy in patients who are exposed to uncontrollable stressful life events.

## Figures and Tables

**Table 1:** Demographic and clinical characteristics stratified by depression group.

	Current-depressed (*n* = 37)	Past-depressed (*n* = 25)	Never-depressed (*n* = 45)	*F* or *X*^2^ or *t*
Gender: women *n* (%)	26 (70.3)	15 (60.0)	29 (64.4)	0.36
Age *M* (SD)	32.62 (16.01)^a^	26.68 (12.20)^ab^	25.49 (11.47)^b^	3.11⁣^∗^
Ethnicity: White *n* (%)^1^	30 (81.1)	18 (72.0)	31 (68.9)	12.26
East or South Asian	4 (10.8)	4 (16.0)	11 (24.4)	
Black	0	2 (8.0)	0	
Other	2 (5.4)	0	1 (2.2)	
Socioeconomic status	3.75 (2.08)	3.68 (1.99)	3.32 (1.88)	0.52
HRSD score	17.38 (6.55)^a^	4.96 (3.81)^b^	0.89 (1.39)^c^	151.00⁣^∗∗∗^
AD score	85.28 (11.02)^a^	64.29 (12.43)^b^	48.96 (9.24)^c^	117.7⁣^∗∗∗^
Age of first depression *M* (SD)	21.27 (11.74)	19.04 (10.11)		0.77
Lifetime episode no. *M* (SD)	2.86 (2.25)	1.52 (0.82)		3.25⁣^∗∗^
Antidepressant: Yes *n* (%)	20 (54.1)	10 (40.0)		0.94
Comorbidity: Yes *n* (%)^2^	20 (54.1)	12 (48.0)		0.30
Agoraphobia	1 (2.7)	0		
Anxiety disorder NOS	2 (5.4)	0		
Eating disorder NOS	0	1 (4.0)		
GAD	7 (18.9)	2 (8.0)		
OCD	1 (2.7)	3 (12.0)		
Panic disorder	2 (5.4)	3 (12.0)		
PTSD	2 (5.4)	1 (4.0)		
Social anxiety disorder	12 (32.4)	6 (24.0)		
Specific phobia	1 (2.7)	0		
Substance abuse	2 (5.4)	0		

⁣^∗^*p* < .05; ⁣^∗∗^*p* < .01; ⁣^∗∗∗^*p* < .001. Letter superscripts represent subgroup differences *p* < .05. Note: HRSD = Hamilton Rating Scale for Depression; AD = Anhedonic Depression subscale in the Mood and Anxiety Symptom Questionnaire; NOS = not otherwise specified; GAD = generalized anxiety disorder; OCD = obsessive-compulsive disorder; PTSD = posttraumatic stress disorder. ^1^Four participants did not report their ethnicity. ^2^Some participants had more than one comorbid diagnosis.

**Table 2:** Descriptive statistics for primary study variables stratified by depression group.

	Current-depressed (*n* = 37)	Past-depressed (*n* = 25)	Never-depressed (*n* = 45)	*F*
Independent events	2.08 (2.66)	1.72 (2.75)	1.69 (2.47)	0.26
Dependent-interpersonal events	0.92 (1.59)	1.40 (1.63)	0.84 (1.40)	1.15
Dependent-non-interpersonal events	1.51 (1.48)	1.16 (1.18)	1.50 (1.96)	0.44
Response bias	0.15 (0.15)	0.16 (0.17)	0.09 (0.21)	1.86
Reward learning	0.06 (0.21)	0.14 (0.22)	0.11 (0.29)	0.83

Note: All *p*s > .16.

**Table 3:** Parameter estimates for the models predicting reward learning from depression group, stressful life events, and their interaction.

	*b*	SE	*t*	*p*	*F*(11, 95)	Adjusted *R*^2^
Overall model				.15	1.48	0.15
Intercept	0.12	0.06	2.04	.04		
Depression group (CD vs. ND)	-0.07	0.09	-0.79	.43		
Depression group (PD vs. ND)	-0.01	0.11	-0.10	.92		
Independent events	0.03	0.02	1.64	.10		
Dependent-non-interpersonal events	-0.02	0.02	-1.12	.26		
Dependent-interpersonal events	-0.02	0.03	-0.74	.46		
CD vs. ND∗independent events	-0.05	0.02	-2.33	.02		
PD vs. ND∗independent events	<0.001	0.02	0.17	.87		
CD vs. ND∗noninterpersonal events	0.08	0.04	2.12	.04		
PD vs. ND∗noninterpersonal events	0.02	0.05	0.35	.72		
CD vs. ND∗interpersonal events	<0.001	0.04	0.02	.98		
PD vs. ND∗interpersonal events	0.01	0.04	0.30	.77		

Note: CD = current-depressed; ND = never-depressed; PD = past-depressed.

**Table 4:** Parameter estimates for the model predicting response bias from depression and stressful life events.

	*b*	SE	*t*	*p*	*F*(5, 101)	Adjusted *R*^2^
Overall model				.05	2.29	0.06
Depression group (CD vs. ND)	0.06	0.04	1.42	.16		
Depression group (PD vs. ND)	0.07	0.04	1.54	.13		
Independent events	0.02	0.01	2.70	.01		
Dependent-interpersonal events	<-0.001	0.01	-0.25	.81		
Dependent-non-interpersonal events	<-0.001	0.01	-0.20	.84		

Note: CD = current-depressed; ND = never-depressed; PD = past-depressed.

## Data Availability

The raw data used to support the findings of this study are available from the corresponding author upon request.

## Supplementary Materials

Supplementary Materials The results of analyses with the inclusion of study (1 vs. 2) as a covariate are presented in the Supplementary Materials (see Supplemental Tables 1 and 2). Additionally, the descriptive statistics of the number and percentage of participants in each depression group who reported at least one event of each type are provided in the Supplemental Material (see Supplemental Table 3).
