# Supplementary material for: Stressful Life Events and Reward Processing in Adults: Moderation by Depression and Anhedonia
Source: Depress Anxiety. 2024 Feb 3;2024:8853631. doi: 10.1155/2024/8853631 (PMC11918537; doi:10.1155/2024/8853631)
Supplement: Supplementary Materials — The results of analyses with the inclusion of study (1 vs. 2) as a covariate are presented in the Supplementary Materials (see Supplemental Tables 1 and 2). Additionally, the descriptive statistics of the number and percentage of participants in each depression group who reported at least one event of each type are provided in the Supplemental Material (see Supplemental Table 3). [file 8853631.f1.docx]

Table 1

*Parameter Estimates for the Models Predicting Reward Learning from Depression Group, Stressful Life Events, and their Interaction Controlling for Study*

|  | *b* | *SE* | *t* | *p* | *F*(12, 94) | *adjusted R^2^* |
| --- | --- | --- | --- | --- | --- | --- |
| Overall model |  |  |  | .20 | 1.35 | 0.04 |
| Intercept | 0.11 | 0.07 | 1.46 | .15 |  |  |
| Study (1 vs. 2) | 0.01 | 0.05 | 0.27 | .79 |  |  |
| Depression Group (CD vs. ND) | -0.07 | 0.09 | -0.81 | .43 |  |  |
| Depression Group (PD vs. ND) | -0.01 | 0.11 | -0.06 | .95 |  |  |
| Independent Events | 0.03 | 0.02 | 1.65 | .10 |  |  |
| Dependent Non-Interpersonal Events | -0.02 | 0.02 | -0.99 | .32 |  |  |
| Dependent Interpersonal Events | -0.02 | 0.03 | -0.71 | .48 |  |  |
| CD vs. ND * Independent Events | -0.05 | 0.02 | -2.29 | .02 |  |  |
| PD vs. ND * Independent Events | <0.001 | 0.02 | 0.12 | .91 |  |  |
| CD vs. ND * Non-Interpersonal Events | 0.08 | 0.04 | 2.02 | .046 |  |  |
| PD vs. ND * Non-Interpersonal Events | 0.02 | 0.05 | 0.34 | .73 |  |  |
| CD vs. ND * Interpersonal Events | <0.001 | 0.04 | 0.03 | .98 |  |  |
| PD vs. ND * Interpersonal Events | 0.01 | 0.04 | 0.31 | .76 |  |  |

Note: CD = current-depressed; ND = never-depressed; PD = past-depressed.

Table 2

*Parameter Estimates for the Model Predicting Response Bias from Depression and Stressful Life Events Controlling for Study*

|  | *b* | *SE* | *t* | *p* | *F*(6, 100) | *adjusted R^2^* |
| --- | --- | --- | --- | --- | --- | --- |
| Overall model |  |  |  | .01 | 2.83 | 0.09 |
| Study (1 vs. 2) | 0.08 | 0.04 | 2.18 | .03 |  |  |
| Depression Group (CD vs. ND) | 0.04 | 0.04 | 1.03 | .30 |  |  |
| Depression Group (PD vs. ND) | 0.08 | 0.04 | 1.87 | .06 |  |  |
| Independent Events | 0.02 | 0.01 | 3.06 | <.001 |  |  |
| Dependent Interpersonal Events | <0.001 | 0.01 | -0.04 | .97 |  |  |
| Dependent Non-interpersonal Events | <0.001 | 0.01 | 0.30 | .76 |  |  |

Note: CD = current-depressed; ND = never-depressed; PD = past-depressed.

Table 3

*Frequency and Percentage of Participants in Each Group Reporting at Least One Event of Each Type in the 6-Months Prior to the PRT*

|  | Current-depressed (*n* = 37) | Past-depressed (*n* = 25) | Never-depressed (*n* = 45) | χ^2^ |
| --- | --- | --- | --- | --- |
| Independent *n*(%) | 24 (65) | 13 (52) | 23 (51) | 1.88 |
| Dependent non-interpersonal *n*(%) | 26 (70) | 17 (68) | 14 (56) | 3.06 |
| Dependent interpersonal *n*(%) | 12 (32) | 14 (56) | 16 (46) | 1.80 |

Note: PRT = Probabilistic Reward Task. All chi-square tests non-significant, *p*s > .20.
